# A Comparison of Oral Function in Older In- and Outpatients: An Observational Study

**DOI:** 10.3390/ijerph21080995

**Published:** 2024-07-29

**Authors:** Anna K. Eggimann, Leo Badura, Rahel Zehnder, Miriam Koemeda, Ramona Buser, Martin Schimmel

**Affiliations:** 1 Department of Geriatrics, Inselspital, Bern University Hospital, University of Bern, Freiburgstrasse, 3010 Bern, Switzerland; 2 Department of Reconstructive Dentistry and Gerodontology, School of Dental Medicine, University of Bern, 3010 Bern, Switzerland; 3 Medical Faculty, University of Bern, 3012 Bern, Switzerland

**Keywords:** masticatory performance, bite force, supporting zones, oral screening, geriatric

## Abstract

(1) Background: Insufficient data exist regarding oral function among older adults in Europe. Therefore, we aimed to assess and compare oral function between older in- and outpatients and identify predictors of low masticatory performance. (2) Methods: Patients were consecutively recruited from the outpatient center (*n* = 31) and the inpatient geriatric department (*n* = 31) at a tertiary University Hospital in Switzerland in 2023. Assessments on oral function included the total number of intraoral eruptive teeth, number of supporting zones with dentures, maximal bite force (Dental Prescale II, Fuji Film Corp., Tokyo, Japan), and masticatory performance (Hue-Check Gum©, University of Bern, Bern, Switzerland) using a visual (SA1–SA5) and a digital (SD_Hue) scale. The visual and digital assessment of masticatory performance showed a strong correlation (Kendall tau = 0.83). Low masticatory performance was defined as SA-Grade 1–2 (vs. SA3–SA5 as reference). In a multivariate model adjusting for age, sex, and clinical setting, we investigated associations of maximal bite force, few eruptive teeth, and few supporting zones with low masticatory performance. (3) Results: Mean age was 81.9 (standard deviation (sd) 5.2) years, and 62.9% were female. Overall, maximal bite force was 247 N (sd 261). A total of 39 patients (63.9%) had a low masticatory performance, 62.9% a low maximal bite force, and 50% a low number of eruptive teeth (<10). Masticatory performance, number of eruptive teeth, and maximal bite force did not significantly differ between in- and outpatients. The number of supporting zones was significantly higher in outpatients compared to inpatients (median 4, interquartile range (IQR) 4–4; vs. 4, IQR 2–4; *p* = 0.03). In the multivariate model, maximal bite force and a low number of eruptive teeth were independently associated with low masticatory performance (adjusted odds ratio 7.4 (95% CI, 1.8–30.4; *p* < 0.01), and OR 7.8 (95% CI, 1.7–36.4; *p* < 0.01), respectively). (4) Conclusions: Impaired oral function is highly prevalent in both European older in- and outpatients to a similar degree. The association of low masticatory performance with maximal bite force and with a low number of eruptive teeth may indicate that a basic screening should include either of these parameters to identify impaired oral function.

## 1. Introduction

As life expectancy increases, so does the prevalence of impaired oral function among older adults. The life expectancy at birth in Switzerland in 2022 was 85.4 for women and 81.6 years for men, indicating gradual aging of the population over time [1]. Due to the cumulative effect of oral diseases, older individuals may experience a greater incidence of dental problems compared to younger individuals, including issues like tooth loss [2,3].

Oral function has a direct impact on general health status. Patel et al. states that oral health is a key component of overall health and well-being [4]. Numerous studies have consistently demonstrated the significant association of oral function and adverse health outcomes, including conditions like sarcopenia and frailty [5,6,7,8]. Specifically, a Japanese study reported an association of overall muscle strength and physical performance with oral function [9]. Nagamine et al. [10] showed that poor oral function is associated with higher risk of loss of independence or death in generally healthy older adults. Similarly, a systematic review revealed that poor oral health status was associated with functional disability, poor quality of life, and mortality [11].

Therefore, it is crucial to promptly identify oral health issues and implement targeted therapeutic interventions to prevent these adverse outcomes [12]. Studies have shown that masticatory performance not only improves overall well-being but that it also plays a crucial role in preserving cognitive functions [13]. Moreover, a recent systematic review reported masticatory function being associated with systemic diseases such as stroke, chronic pulmonary disease, and carotid atherosclerosis [14]. These findings suggest that maintaining an efficient masticatory function could have substantial implications for general health.

Assessment on oral function can be conducted using objective clinical examinations, which involve evaluating factors like masticatory performance [15]. Alternatively, subjective assessments can be carried out using specialized questionnaires designed to measure masticatory performance accurately [16,17]. However, assessment on oral function is not routinely included as a standard component in the geriatric assessment across various clinical settings, calling for urgent action [4]. A recent systematic review summarized that while oral function is a well-acknowledged field in Japan [18], observational data for older patients in Europe are scarce.

To the best of our knowledge, data are insufficient on oral function among European older adults in both in- and outpatient settings. Therefore, we aimed to investigate and compare oral function between older in- and outpatients and to identify predictors of impaired masticatory performance.

## 2. Materials and Methods

In this observational study, patients were recruited from two distinct clinical settings. Older outpatients were recruited from August 2023 to September 2023 at the Department for Reconstructive Dentistry and Gerontology (School of Dental Medicine, University of Bern, Bern, Switzerland). Inclusion criteria required outpatients to be aged 70 years or older. Outpatients undergoing dental hygiene services following rehabilitation with dental prostheses on implants and/or natural teeth were included.

Older inpatients were consecutively recruited at the geriatric rehabilitation unit at the University Hospital Bern in October 2023. To be included in the study, the following admission criteria had to be met: (1) age 70 years and older, (2) direct transfer from acute care hospital, (3) living in the community (i.e., not in a nursing home) prior to acute care hospital admission, (4) potential for functional improvement, and (5) discharge home following inpatient rehabilitation.

All patients consecutively admitted for geriatric inpatient rehabilitation, meeting the specified criteria, were included. No patients were excluded from this study. Similarly, all patients from the outpatient service were investigated.

This study was conducted according to the research regulations pertaining to human subjects’ health-related data (Swiss Ethics committee Req-2023-00665).

A trained clinical assessor (R.Z. or L.B.) performed the following assessment on oral functions using a standardized protocol.

A dental examination was performed counting the number of existing intraoral erupted teeth, excluding residual roots, by visual inspection. The Eichner classification [19] was determined with dentures in place, whenever available. In short, the Eichner classification features 10 categories assessing the intermaxillary contact in four occlusal supporting zones (in the premolar and molar regions). The number of supporting zones was then derived from the Eichner classification (A1–A3:four supporting zones; B1: three supporting zones; B2: two supporting zones; B3: one supporting zone; B4–C3: zero supporting zones).

To measure the maximum occlusal bite force, the Dental Prescale II system (Fuji Film Corp., Tokyo, Japan) was used. This system consists of a pressure-sensitive film with a thickness of approximately 150 µm. If any dentures were present, the patient was asked to wear them during the assessment. The U-shaped pressure-sensitive foil was placed on the dental arch/dentures in the lower jaw so that the entire set of teeth was covered. The patients were then instructed to clench their teeth as hard as they could and hold the position for three seconds before letting go slowly. After use, the foil was disinfected with an alcohol wipe. Analyses of the Dental Prescale II foil were made with a Bite Force Analyzer (Bite Force Analyzer 2.1.1, GC, Fuji Film Corp, Tokyo, Japan), an image analysis software which uses Dental Prescale II scans from an optical image scanner (Epson Perfection V600 Photo, Epson, Nagano, Japan). A pressure filter function was added to the improved Bite Force Analyzer, which automatically excluded areas of red coloration that were likely generated by a force other than occlusal contact at maximum occlusal force15. Various parameters could be determined with the software, such as (1) maximum occlusal bite force (in Newton), (2) bite force display area (mm^2^), (3) average pressure (in megapascal), (4) maximum pressure (in megapascal), and (5) force distribution of right and left (in percentage).

Masticatory performance was assessed with a two-color mixing ability test. A specially prepared chewing gum with two different colors (Hue-Check Gum©, University of Bern, Switzerland) was used as the test food due to the ability to measure mixing with this [20]. Participants were asked to chew the gum for 20 cycles. The samples were then dried and placed in a clear plastic bag and pressed manually to 1 mm thickness. They were then subjected to visual assessment (SA1–SA5, Orophys subjective assessment scale). The Orophys subjective assessment scale [21,22] has the following five categories.

- SA1 chewing gum not mixed, impressions of cusps or folded once.
- SA2 large parts of chewing gum unmixed.
- SA3 bolus slightly mixed, but bits of unmixed original color.
- SA4 bolus well mixed, but color not uniform.
- SA5 bolus perfectly mixed with uniform color.

Using a flatbed scanner (Epson Perfection V30, Epson, Nagano, Japan), both sides of the pressed gum were scanned. The resulting images were analyzed using the freely available software (ViewGum©, Version 4.1.2.1, https://dhal.com/dowload.htm accesssed on 15 January 2024), which calculates the variance of the Hue (SD_Hue). The variance of the Hue (SD_Hue) is a measure of bolus-mixing [20], whereas a better mixture between the two different colored chewing gums corresponds to a good masticatory performance and is reflected in a low SD_Hue. Conversely, the greater the SD_Hue, the worse the mixing of the chewing gums. Low masticatory performance was defined as SA-Grade 1–2 (vs. SA3–SA5 as reference).

### Statistical Analyses

Mean (standard deviation), median (interquartile range), and number (proportion) for continuous, ordinal, and categorical variables were calculated. The *t*-test was used to compare the mean differences in continuous variables, and chi2 was used for categorical variables between in- and outpatients. The a priori sample size for this study was set at 30 outpatients and 30 inpatients, respectively, allowing us to compare the main characteristics of oral function between the two groups. Kendall’s tau test was performed to compare the correlation of the visual and digital scale of masticatory performance. As an exploratory endpoint, a logistic multivariate model was fitted adjusting for age (80+ years vs. <80 years), sex (female vs. male), and setting (in- vs. outpatients). Statistical analyses were performed using Stata (StataCorp LLC, College Station, TX, USA, Version 16). A *p*-value of <0.05 was considered statistically significant. 

## 3. Results

A total of 62 patients aged ≥ 70 years (mean 81.9 ± 5.2 years) were examined, including 31 inpatients (21 females and 10 males) and 31 outpatients (18 females and 13 males). Characteristics are shown in Table 1 stratified for in- and outpatients. Overall, 63.9% of patients had low masticatory performance with dentures, 62.9% had low maximal bite force, and 50% had a low number of eruptive teeth (<10). There was no significant difference in age between in- and outpatients. Similarly, masticatory performance, eruptive teeth, and maximal bite force did not significantly differ between in- and outpatients. In contrast, the number of supporting zones was significantly higher in outpatients compared to inpatients (*p* = 0.03). Clinical characteristics stratified by sex are shown in the Supplementary Materials (Tables S1 and S2).

**Figure 1:**
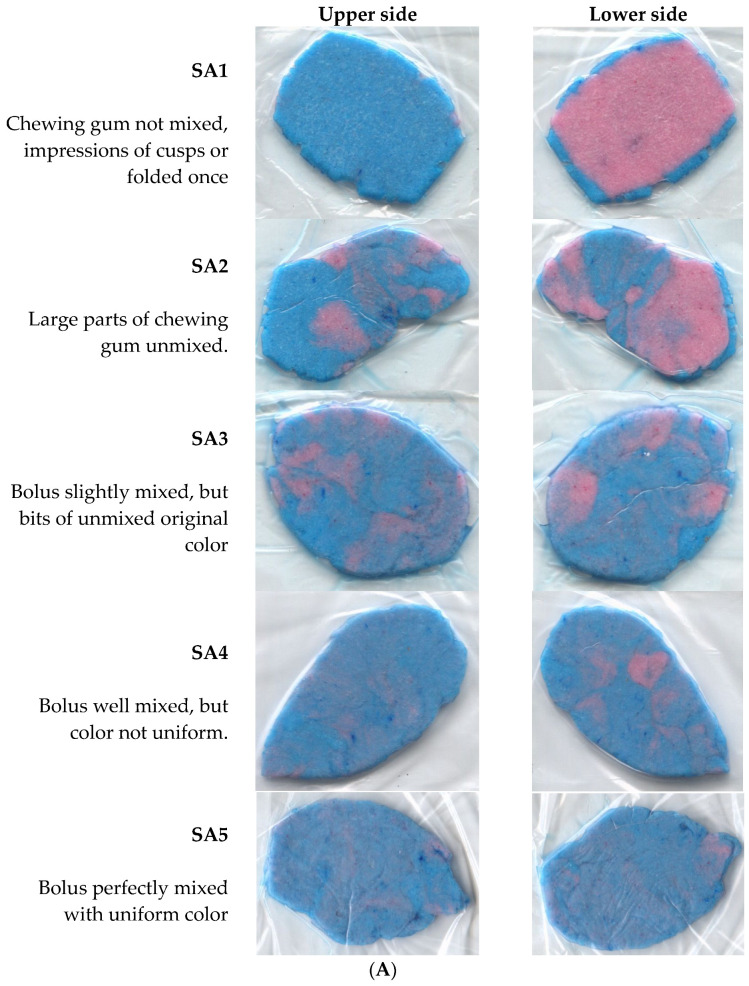

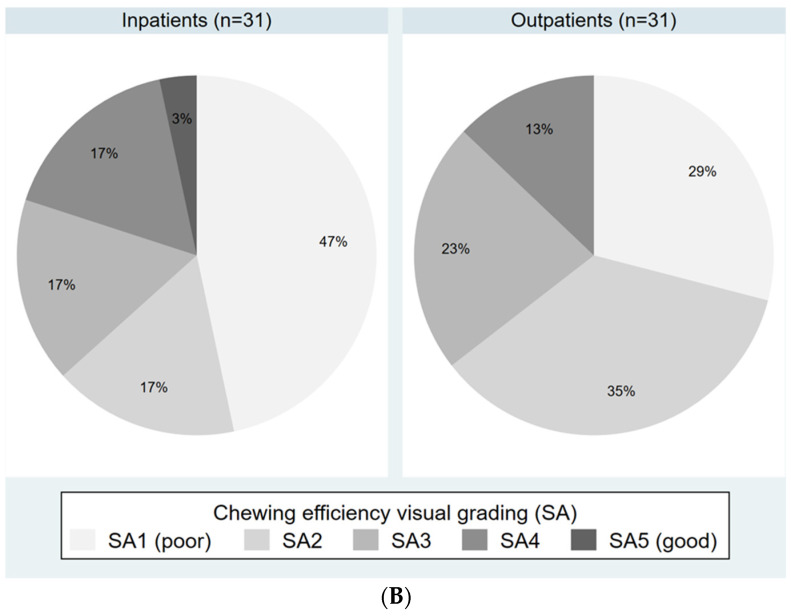
(**A**) Visual assessment of masticatory performance displaying pictures of patients by subjective assessment categories (SA1–SA5). (**B**) Masticatory performance based on visual grading (SA) stratified by clinical setting. Abbreviations: SA; Orophys subjective assessment scale.

Figure 1A displays patient examples of the visual grading of masticatory performance. Figure 1B shows pie charts of masticatory performance based on visual grading (SA) stratified by clinical setting. Low masticatory performance was defined as SA-Grade 1–2 (vs. SA3–SA5 as reference). Numbers of supporting zones stratified by clinical setting can be found in the Supplementary Materials (Figure S1). 

**Figure 2:**
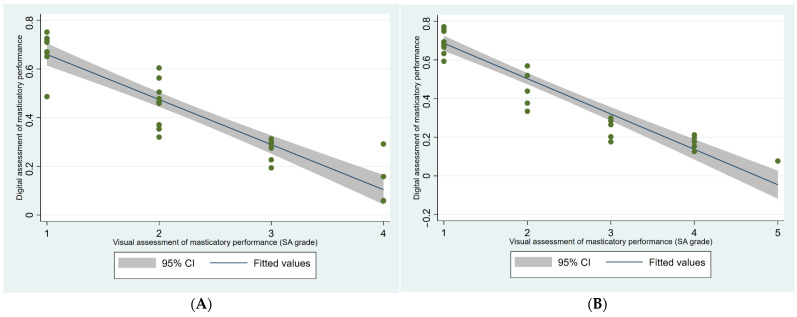
(**A**) Correlation of masticatory performance based on digital scan scale and visual scale among older outpatients (*n* = 31) r = 0.83 (Kendall’s tau-b correlation). (**B**) Correlation of masticatory performance based on digital scan scale and visual scale among older inpatients (*n* = 30) r = 0.83 (Kendall’s tau-b correlation).

Figure 2A,B show the correlation of masticatory performance based on digital scan scale and visual scale among older outpatients (*n* = 31, panel A) and older inpatients (*n* = 30, panel B). Visual and digital assessment of masticatory performance showed a strong correlation (Kendall tau = 0.83, for inpatients as well as outpatients).

In the multivariate model, maximal bite force and a low number of eruptive teeth were independently associated with low masticatory performance (Table 2; adjusted odds ratio 7.4 (95% CI, 1.8–30.4; *p* < 0.01), and OR 7.8 (95% CI, 1.7–36.4; *p* < 0.01), respectively). In contrast, a low number of supporting zones with dentures was not significantly associated with low masticatory performance (adjusted OR 2.2 (95% CI 0.5–9.7; *p* = 0.3).

## 4. Discussion

We found a high prevalence of impaired oral function in both older in- and outpatients. Overall, masticatory performance and maximal bite force were found to be low but comparable across clinical settings. Moreover, maximal bite force and a low number of eruptive teeth were independently associated with low masticatory performance.

To our knowledge, this is the first study to compare older outpatients and inpatients in terms of oral function. Therefore, direct comparisons with other studies are not feasible.

Overall, the prevalence of impaired oral function was high in both older in- and outpatients. While half of patients in our study had a low number of eruptive teeth (<10), a Swedish study found 44% of hospitalized trauma patients with <20 teeth [23]. Moreover, we found 63.9% of patients to have low masticatory performance. Similarly, Okada et al. [16] described 47.8% of community-dwelling older adults having a low masticatory performance. In our study, oral function parameters were comparable across clinical settings, but the number of supporting zones was significantly higher in outpatients compared to inpatients. A possible explanation for this finding may be that outpatients are more attentive to oral health being recruited after outpatient dental treatment whereas inpatients are a mixed sample of patients not necessarily seeing a dentist on a regular basis.

We further found that maximal bite force and a low number of eruptive teeth were independently associated with low masticatory performance. This is in line with a systematic review showing that the number of natural/remaining teeth has the strongest positive association with masticatory performance, but with a very low quality of evidence [24]. Similarly, a recent scoping review found that tooth loss is the most common risk factor for masticatory dysfunction [15]. Komiyama et al. [25] even described maximum occlusal force and number of teeth best predicting incident functional disability among Japanese community-dwelling older adults. Of note, Hatta et al. recently found that occlusal force did not decrease over a three-year period, suggesting that masticatory muscles are less susceptible to aging than other muscles such as tongue-related muscles [26].

We found no significant association of supporting zones with masticatory performance. In contrast, Higashi et al. [27] described an association of occlusal support with masticatory performance. From a pathophysiological perspective, this association is evident. The most reasonable explanation why we did not find this association between occlusal support and masticatory performance is that our sample size was too small to observe a significant effect.

We further found a strong correlation between the digital and visual assessment of masticatory performance. Accordingly, Schimmel et al. [20,28] concluded that visual assessment may be sufficient for daily clinical practice to assess masticatory performance. Our results may indicate that visual assessment may be an easy rapid starting assessment to evaluate masticatory performance in older hospitalized patients.

There are several limitations to this study. First, study sample size is limited, so there is a potential risk of underpowering the analysis. Nevertheless, the data presented provide a starting point for future research in this underrepresented field in Europe. Second, while the study is considered valid for these study settings in Switzerland, data may not be representative of other clinical settings and other countries. Third, we measured key parameters of oral function among older patients and, therefore, the results may not apply to other assessments. However, the methodology of the measurements is solid as we applied validated and commonly implemented assessments on oral function.

Our study’s findings have significant implications for both research and clinical practice. The high prevalence of patients experiencing poor oral function highlights the need for a routine screening in the inpatient and outpatient setting. Only through such screening can interventions be implemented to prevent adverse outcomes. Although oral function assessment is more commonly performed in outpatient dental services, it is not yet a routine practice. Furthermore, in the inpatient setting of geriatric care, there is a complete absence of routine assessment for oral function. The attention of clinicians needs to be drawn to masticatory function to patients who suffer from physical dysfunction [14]. A recent Japanese study highlighted a potential relationship between intrinsic capacity, such as locomotion and oral function [29]. Robinson et al. [30] suggested addressing frailty by implementing oral health measures. A randomized trial demonstrated that masticatory muscle training program as a targeted intervention significantly improved masticatory performance in people with cognitive impairment or dementia [31].

Our study experiences indicate that clinically trained assessors who conduct standard geriatric assessments are capable of performing oral function screenings. Based on our finding of the multivariate model, a simple and feasible approach would be to initiate screening in medical settings with a minimal set of one assessment on oral function. Lahoud et al. suggested implementing the chewing gum method as a standard to encourage clinicians to routinely measure oral function. This approach would also ensure feasibility in daily clinical practice. The observations of our study should be interpreted as a starting point for addressing a previously neglected field in older adults. Future research should involve larger study populations to confirm our findings. Additionally, observational cohort studies should explore the associations of oral function with other common diseases in older adults such as sarcopenia and frailty in both outpatient and inpatient settings.

## 5. Conclusions

In conclusion, impaired oral function is highly prevalent in both older in- and outpatients to a similar degree. The association of low masticatory performance with maximal bite force and with a low number of eruptive teeth may indicate that a basic screening should include either of these parameters to identify impaired oral function.

## Figures and Tables

**Table 1:** Clinical characteristics of patients stratified by clinical setting (*n* = 62).

Characteristic	Overall (*n* = 62)	Outpatients (*n* = 31)	Inpatients (*n* = 31)	*p*-Value
Age, mean (sd)	81.9 (5.2)	80.7 (4.3)	83.1 (5.9)	0.08
Female sex, *n* (%)	39 (62.9)	18 (58.1)	21 (67.7)	0.43
Eruptive teeth (*n*), mean (sd)	10.56 (9.2)	9.1 (7.9)	12.0 (10.3)	0.21
Dentures upper jaw, *n* (%)				n.a.
-Natural teeth	15 (24.2)	4 (12.9)	11 (35.5)	
-Partial dentures	22 (35.5)	17 (54.8)	5 (16.1)	
-Total dentures	25 (40.3)	10 (32.3)	15 (48.4)	
Dentures lower jaw, *n* (%)				n.a.
-Natural teeth	23 (37.1)	6 (19.4)	17 (54.8)	
-Partial dentures	28 (45.2)	23 (74.2)	5 (16.1)	
-Total dentures	11 (17.7)	2 (6.4)	9 (29.0)	
Bite force (N), mean (sd)	247 (261)	212.2 (178.6)	283.1 (323.5)	0.29
Bite force display area (mm^2^), mean (sd)	6.5 (5.9)	5.6 (4.1)	6.2 (4.8)	0.60
Average pressure (N/mm^2^), mean (sd)	32.8 (11.6)	34.7 (11.0)	31.0 (12.2)	0.22
Maximum pressure (N/mm^2^), mean (sd)	77.7 (38.3)	79.5 (35.6)	76.2 (37.3)	0.72
Force distribution right side (%), mean (sd)	47.9 (24.5)	49.3 (22.5)	46.6 (26.7)	0.67
Force distribution left side (%), mean (sd)	45.6 (24.2)	47.6 (22.4)	43.7 (26.1)	0.53
Supporting zones with dentures (*n*), median (IQR)	4 (3–4)	4 (4–4)	4 (2–4)	0.03
Eichner classification with dentures, median (IQR)	A1 (A1–B1)	A1 (A1–A1)	A3 (A1–B2)	<0.01
Masticatory performance, visual scale, median (IQR)	SA2 ^b^ (SA1–SA3)	SA2 (SA1–SA3)	SA2 ^a^ (SA1–SA3)	0.59
Masticatory performance, digital scale (VoH), mean (sd)	0.46 ^b^ (0.22)	0.44 (0.20)	0.48 ^a^ (0.25)	0.49

Abbreviations: sd = standard deviation; VoH = variance of the Hue; IQR = interquartile range; n.a., not applicable; ^a^ *n* = 30 (*n* = 1 missing); ^b^ *n* = 61 (*n* = 1 missing).

**Table 2:** Predictors of low masticatory performance ^a^ based on the visual assessment scale (*n* = 62).

Oral Health Variable	N (%) Inpatients with Normal Masticatory Performance	N (%) Inpatients with Low Masticatory Performance	Crude OR (95% CI)	*p*-Value	Adjusted OR (95% CI) ^b^	*p*-Value	Full Model OR (95%CI) ^c^	*p*-Value
Maximum bite force								
Normal (>265 N)	15 (68.2%)	7 (18.0%)	Reference		Reference			
Low (≤265 N)	7 (31.8%)	32 (82.1%)	9.8 (2.9–33.0)	<0.01	9.6 (2.8–32.7)	<0.01	7.4 (1.8–30.4)	<0.01
Eruptive teeth								
≥10	18 (81.8%)	12 (30.8%)	Reference		Reference			
0–9	4 (18.2%)	27 (69.2%)	10.1 (2.8–36.4)	<0.01	9.6 (2.7–35.1)	<0.01	7.8 (1.7–36.4)	<0.01
Supporting zones with dentures								
3–4	19 (86.4%)	30 (61.2%)	Reference		Reference			
Low (0–2)	3 (13.6%)	39 (63.9%)	1.9 (0.5–7.9)	0.38	2.2 (0.5–9.7)	0.30	4.9 (0.7–34.7)	0.11

Abbreviations: OR, odds ratio; CI, confidence interval; N, Newton. ^a^ Low masticatory performance defined as SA1–SA2 vs. reference standard of SA3–SA5. ^b^ Adjusted for age (≥80 years vs. 80 years), sex, and setting (in- vs. outpatients). ^c^ Full model including variables maximum bite force, eruptive teeth, supporting zones with dentures adjusted for age (≥80 years vs. 80 years), sex, and setting (in- vs. outpatients).

## Data Availability

All data are provided in the manuscript and Supplementary Materials.

## Supplementary Materials

The following supporting information can be downloaded at: https://www.mdpi.com/article/10.3390/ijerph21080995/s1, Figure S1: Pie charts displaying number of supporting zones stratified by clinical setting; Table S1: Characteristics of older outpatients stratified by sex (*n* = 31); Table S2: Characteristics of older inpatients stratified by sex (*n* = 31).
