# Supplementary material for: A Comparison of Oral Function in Older In- and Outpatients: An Observational Study
Source: Int J Environ Res Public Health. 2024 Jul 29;21(8):995. doi: 10.3390/ijerph21080995 (PMC11353424; doi:10.3390/ijerph21080995)
Supplement: Supplementary file 1 [file ijerph-21-00995-s001.zip › ijerph-3103279-supplementary.pdf]

International Journal of
Environmental Research and Public Health

MDPI

Supplementary Materials

A Comparison of Oral Function in Older In- and Outpatients: An Observational Study

Anna K. Eggimann [1,*], Leo Badura [2], Rahel Zehnder [1,3], Miriam Koemeda [1], Ramona Buser [2] and Martin Schimmel [2]

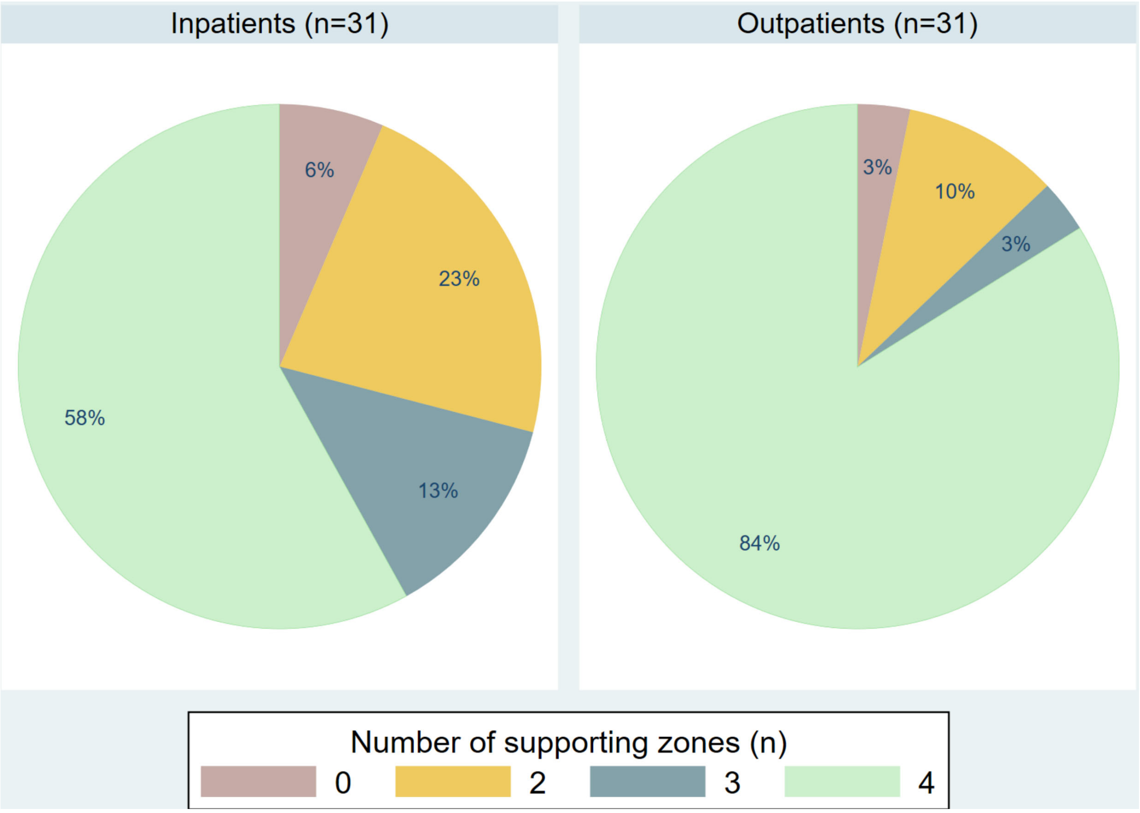

Figure S1. Pie charts displaying number of supporting zones stratified by clinical setting.

Table S1. Characteristics of older outpatients stratified by sex (*n* = 31).

Characteristic	Female (*n* = 18)	Male (*n* = 13)	P-value
Age, mean (sd)	80.1 (4.1)	81.6 (4.5)	0.34
Eruptive teeth (n), mean (sd)	10.4 (8.4)	7.2 (7.1)	0.27
Bite force (N), mean (sd)	233.5 (211.7)	182.8(121.1)	0.44
Bite force display area (mm^2), mean (sd)	6.2 (4.8)	4.7 (2.6)	0.32
Average pressure (N/mm^2), mean (sd)	34.1 (9.6)	35.4 (13.1)	0.75
Maximum pressure (N/mm^2), mean (sd)	76.2 (37.3)	84.1 (34.1)	0.95
Force distribution right side (%), mean (sd)	46.5 (18.6)	53.1 (27.4)	0.43
Force distribution left side (%), mean (sd)	53.5 (18.6)	39.3 (25.2)	0.08
Supporting zones with dentures (n), median (IQR)	3.9 (0.47)	3.3 (1.25)	0.07
Eichner-classification with dentures, median (IQR)	A1 (A1–A1)	A1 (A1–B1)	0.53
Masticatory performance, visual scale, median (IQR)	SA2 (SA1–SA3)	SA2 (SA2–SA3)	0.47
Masticatory performance, digital scale (SD_Hue), mean (sd)	0.45 (0.20)	0.43 (0.22)	0.79

Abbreviations: sd= standard deviation; VoH = Variance of the Hue; IQR = interquartile range.

Table S2. Characteristics of older inpatients stratified by sex (*n* = 31).

Characteristic	Female (*n* = 21)	Male (*n* = 10)	P-value
Age, mean (sd)	84.0 (5.8)	81.3 (6.1)	0.24
Eruptive teeth (n), mean (sd)	12.5 (9.8)	11.0 (11.9)	0.71
Bite force (N), mean (sd)	263.8 (295.8)	323.8 (389.7)	0.64
Bite force display area (mm^2), mean (sd)	7.0 (6.5)	8.3 (9.2)	0.65
Average pressure (N/mm^2), mean (sd)	30.6 (12.0)	31.8 (13.1)	0.80
Maximum pressure 1N/mm^2), mean (sd)	76.2 (42.1)	75.1 (42.2)	0.95
Force distribution right side (%), mean (sd)	46.2 (26.6)	47.5 (28.3)	0.90
Force distribution left side (%), mean (sd)	44.3 (26.3)	42.5 (27.3)	0.86
Supporting zones with dentures (n), median (IQR)	4 (2-4)	4 (4-4)	0.01
Eichner-classification with dentures, median (IQR)	B1 (A1–B2)	A2 (A1–A2)	0.03
Masticatory performance, visual scale, median (IQR)	SA2* (SA1*–SA3*)	SA1 (SA1–SA3)	1.0
Masticatory performance, digital scale (VoH), mean (sd)	0.46* (0.26*)	0.51 (0.23)	0.61

Abbreviations: sd= standard deviation; VoH = Variance of the Hue; IQR = interquartile range; **n* = 20.
